# Psychosis risk for lesbian, gay, and bisexual individuals: systematic review and meta-analysis

**DOI:** 10.1017/S0033291724002253

**Published:** 2024-10

**Authors:** Jean-Paul Selten, Hussam Alrashed, Hans Oh, Gabriëlla A. M. Blokland

**Affiliations:** 1 Department of Psychiatry and Neuropsychology, Faculty of Health, Medicine, and Life Sciences, Mental Health and Neuroscience Research Institute, Maastricht University, Maastricht, The Netherlands; 2 Faculty of Life Sciences & Medicine, King's College London, London, UK; 3 St. John's Institute of Dermatology, Guy's and St Thomas’ Hospital NHS Foundation Trust, London, UK; 4 Suzanne Dworak Peck School of Social Work, University of Southern California, Los Angeles, CA, USA

**Keywords:** bisexuality, discrimination, homosexuality, meta-analysis, psychosis, psychotic disorder, schizophrenia, sexual minority status, social defeat, systematic review

## Abstract

The social defeat hypothesis posits that low status and repeated humiliation increase the risk for psychotic disorders (PDs) and psychotic experiences (PEs). The purpose of this paper was to provide a systematic review of studies on risk of PDs and PEs among lesbian, gay, or bisexual (LGB) people and a quantitative synthesis of any difference in risk. PubMed, PsycINFO, Embase, and Web of Science were searched from database inception until January 30, 2024. Two independent reviewers assessed the eligibility and quality of studies, extracted effect sizes, and noted the results of mediation analyses. Using a random effects model we computed pooled odds ratios (ORs). Preferred Reporting Items for Systematic Reviews and Meta-Analyses guidelines were followed. The search identified seven studies of PDs and six of PEs. As for PDs, the unadjusted (2.13; 95% confidence interval 0.72–6.34) and covariate-adjusted pooled OR (2.24; 1.72–3.53) were not significantly increased for LGB individuals. After exclusion of a study of limited quality, both the unadjusted pooled OR (2.77; 1.21–6.32) and the covariate-adjusted pooled OR (2.67; 1.53–4.66) were significantly increased. The pooled ORs were increased for PEs: unadjusted, pooled OR = 1.97 (1.47–2.63), covariate-adjusted, pooled OR = 1.85 (1.50–2.28). Studies of PE that examined the mediating role of several variables reported that the contribution of drug abuse was small compared to that of psychosocial stressors. The results of a study in adolescents suggested a protective effect of parental support. These findings suggest an increased psychosis risk for LGB people and support the social defeat hypothesis.

## Introduction

In a recent survey of 30 countries, approximately 9% of respondents identified as lesbian, gay, or bisexual (LGB+), though prevalence varied across countries (https://www.ipsos.com/en/pride-month-2023-9-of-adults-identify-as-lgbt). However, sexual orientation is difficult to estimate, especially in countries where anything other than heterosexuality is prohibited (Pachankis & Bränström, 2019). Furthermore, reports about sexual identity, sexual preference, and sexual behavior often do not align (Patela et al., 2006). There can be no doubt, however, that homophobia and heterosexism cause a lot of stress among LGB people.

People exposed to social stressors are at an increased risk of developing mental health problems and an increasing body of evidence indicates that these stressors are also important in the etiology of schizophrenia spectrum or psychotic disorders (PDs) (e.g. Kirkbride et al., 2024; Selten & Cantor-Graae, 2005; Selten & Ormel, 2023). This evidence includes various research findings, including increased risks for subjects with a childhood trauma (Varese et al., 2012), the presence of any psychiatric disorder in childhood or adolescence (Maibing et al., 2015), a low IQ (Khandaker, Barnett, White, & Jones, 2011), a disadvantaged ethnic minority status (Mirza et al., 2022; Petrović-van der Deen et al., 2020; van der Ven et al., 2024), a hearing impairment (Linszen, Brouwer, Heringa, & Sommer, 2016), or gender dysphoria (e.g. Termorshuizen, de Vries, Wiepjes, & Selten, 2023).

According to the social defeat hypothesis of psychosis the combination of low status, repeated experiences of humiliation, and a poor homeostatic control of dopamine neurons in the midbrain and dorsal striatum lead to increased striatal dopamine activity and thereby to an increased risk of psychosis (Selten & Ormel, 2023). Psychosis has been conceptualized as a continuum between normality and PD, such that about 7% of the adult population has psychotic experiences (PEs), without necessarily crossing the clinical threshold for PD (Linscott & van Os, 2013). Given that risk factors for PD overlap with those for PEs, the social defeat hypothesis would also lead us to expect an increased risk of PEs for sexual minorities.

In this study, we focus on LGB people. The aims of this study were to provide (i) a systematic review of studies on risk of PD and (clinical or non-clinical) PEs among LGB people; and (ii) a quantitative synthesis of any difference in risk.

## Methods

This systematic review and meta-analysis were conducted following the Preferred Reporting Items for Systematic Reviews and Meta-Analyses guidelines (Page et al., 2021). The protocol was prospectively registered on August 26th, 2022 in PROSPERO (CRD42022354853). The final search was performed on January 29th, 2024.

### Eligibility

For inclusion in this systematic review, studies were required to (i) compare the presence of PD or PE among homosexual or bisexual individuals relative to heterosexual individuals; (ii) to provide either the odds ratios (ORs) or relative risks (RRs), or the numerators and denominators that would allow for the calculation of such effects. We excluded publications (i) that only reported figures for groups comprising transgender or queer individuals; and (ii) that reported risk for sub-groups of homosexual or bisexual individuals based on organic factors. Examples are samples characterized by the use of illicit drugs or infection with the human immunodeficiency virus.

### Search strategy and data extraction

A detailed description of our search strategy is available in the online Supplementary Methods. We conducted the systematic review using PubMed, Embase, PsycINFO, and Web of Science. The following information was extracted from each article: (1) authors, year of publication; (2) country where the study was performed; (3) age, sex, and ethnicity of study population; (4) sexual orientation (heterosexual, homosexual, or bisexual); (5) additional details about sexual orientation (orientation, identity, partnership, behavior/activity, attraction); (6) numbers of psychotic and non-psychotic individuals in groups of LGBs and heterosexuals or general population; (7) classification system, diagnostic interview, or questionnaire used for assessment of psychosis (PD or PE); (8) diagnosis (schizophrenia, mood disorder with psychotic features, PE, etc.); (9) OR, RR, hazard ratio, incidence rate ratio for homosexual and/or bisexual individuals relative to heterosexual individuals; (10) unadjusted and, when available, covariate-adjusted effect estimate (with 95% confidence interval [CI] or standard error (s.e.) accordingly); (11) covariates (age, sex, or other variables).

**Figure 1.**
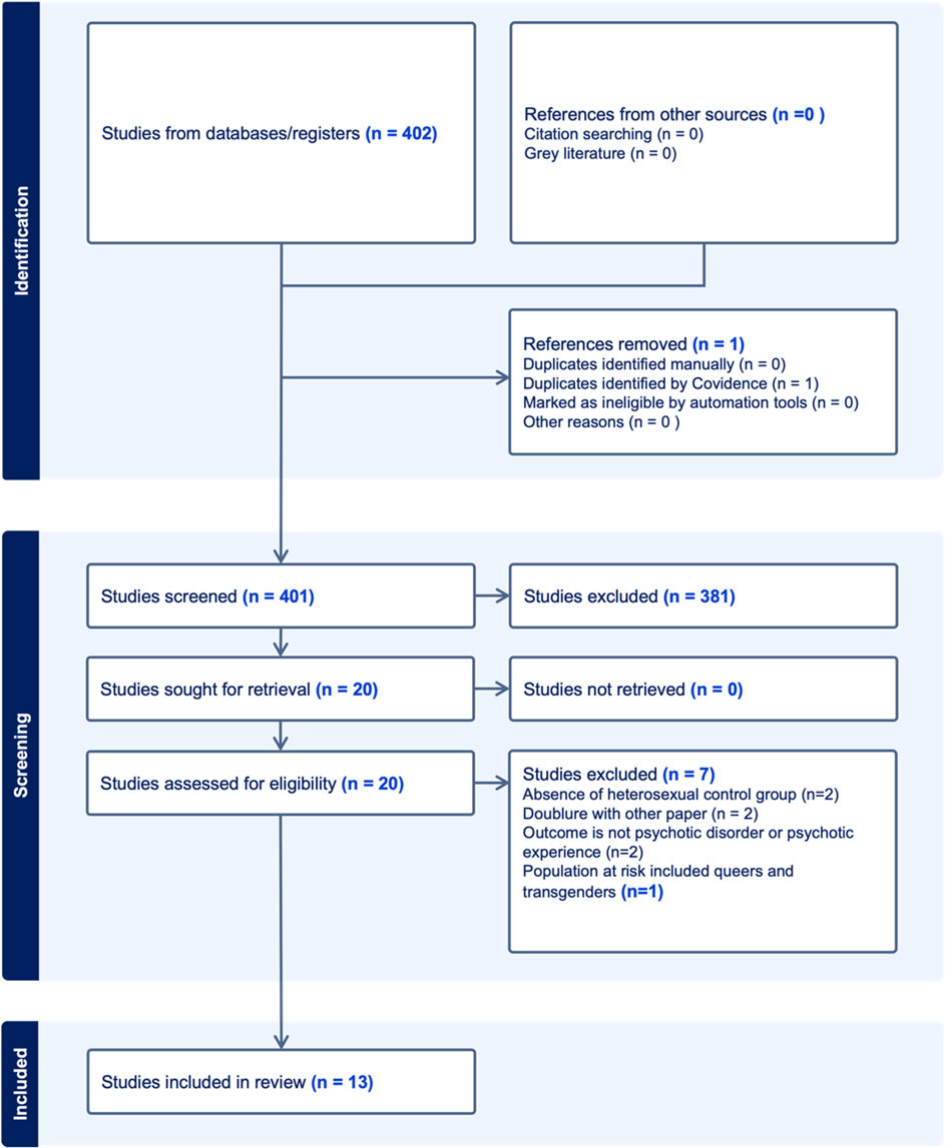
Flow diagram of study selection process.

Two authors (HA and J-PS, or HO and J-PS) independently assessed 401 abstracts of studies for inclusion. The reliability of this decision was fair (Cohen's kappa's 0.44 and 0.69, respectively). HA and J-PS independently reviewed the full text of 20 remaining papers, the reliability of the decision to include or exclude was good (Cohen's kappa = 0.88) (see Fig. 1).

Three authors extracted effect sizes (HA, HO, J-PS). Any differences were discussed in the presence of the last author (GAMB or HO) and agreement was reached through consultation.

### Quality assessment

Since the literature on LGB psychosis risk is still inchoate, we developed our own instrument to assess the quality of each study (see online Supplementary Methods). The quality criteria for studies on PD covered the validity of context (e.g. community survey, case register), sample size, method for assessment of sexual orientation and of PD, classification of PD, inter-rater reliability, and several aspects of statistical analysis. For studies on PEs, the criteria were validity of context (e.g. general population survey, section of population), sample size, method for assessment of sexual orientation, quality of psychosis questionnaire, and statistical analysis.

To check whether the scores obtained correspond to those based on a generally accepted instrument, we also reviewed the studies using the quality assessment tool for observational cohort and cross-sectional studies, developed by the National Institute of Health (NIH; https://www.nhlbi.nih.gov/health-topics/study-quality-assessment-tools).

Two authors (not blind to the identity of the authors) independently assessed the quality of the articles (HA and J-PS). Differences were discussed and reconciled with the opinion of a third author (GAMB or HO) to arrive at a final score.

### Statistical analyses

All statistical analyses were performed using R version 4.3.1 (R Core Team, 2022). If ORs were not reported by the study, then they were calculated using the R package ‘metafor’ (Viechtbauer, 2010).

#### Meta-analyses

Meta-analyses were performed if there were at least two independent cohorts that yielded ORs. Pooled ORs (pooled OR_RE_) were estimated using the R package ‘metafor’ v4.2.0 (Viechtbauer, 2010). In view of the large differences between the pertinent investigations we considered Random Effects (RE) models. RE estimates were based on the restricted maximum-likelihood method. s.e.s and CIs were calculated as suggested by Knapp and Hartung (2003), because this procedure has much more appropriate false-positive rates than the standard approach. In addition to the standard reported *p* values, *p* values were also calculated using a permutation test approach based on Monte Carlo simulation (1000 permutations), which results in more accurate *p* values compared with standard methods, particularly if the number of studies in a model is small.

The proportion of effect size variation that can be attributed to differences between studies (heterogeneity) was calculated using Cochran's *Q*-test and presented using *I*^2^. In addition to the pooled ORs and 95% CIs, the number of included effect sizes (*k*) is reported for each meta-analytic comparison.

Prior to the overall meta-analyses, the covariate-adjusted OR estimates of Bolton and Sareen (2011) were meta-analyzed to generate an overall estimate for males and females combined, and homosexual and bisexual individuals combined, as this study only reported within-subgroup estimates. Likewise, as Lu, Qing, Tu, and Liu (2023) reported only separate results for homosexuals and bisexuals, we conducted a meta-analysis to produce an overall effect estimate for LGBs.

Two studies did not identify a single homosexual or bisexual individual with a PD (Currier et al., 2015; Skerrett, Kõlves, & De Leo, 2014). It is difficult, then, to compute an OR because one cannot divide by zero. Thus, to obtain an OR, we replaced zero with one. The covariate-unadjusted OR thus calculated was used for the meta-analysis of both covariate-unadjusted and covariate-adjusted pooled ORs.

Two studies reported results for sexual minorities (Oh, 2021; Oh et al., 2022). Since this concept includes individuals other than LGB people, separate results for LGBs were obtained from the first author and used here.

Two studies, one on PDs and another on PEs, used two different, but overlapping definitions of LGB status, namely sexual orientation and sexual activity (Gevonden et al., 2014; Qi, Palmier-Claus, Simpson, Varese, & Bentall, 2020). For the overall meta-analysis we used the definition which was associated with the largest number of individuals (i.e. sexual orientation). One study (Post, Veling, & GROUP Investigators, 2021) made a mistake in the calculation of the ORs. We contacted the senior author, who corroborated the error and provided the corrected OR. (The pertinent e-mail is shown in other online Supplementary materials).

Meta-analyses were performed using unadjusted ORs and covariate-adjusted ORs, which resulted in unadjusted pooled ORs and in covariate-adjusted pooled ORs, respectively.

#### Additional analyses

We conducted additional meta-analyses for (i) homosexual individuals *v*. bisexual individuals and (ii) males *v*. females, if at least two studies reported data for these subgroups.

Likewise, if at least two studies reported data, we conducted additional meta-analyses for (i) hallucinations and (ii) delusions.

#### Publication bias

Publication bias was tested by visually assessing the funnel plot for asymmetry and by performing Egger's test (Egger, Davey Smith, Schneider, & Minder, 1997) using the R package ‘metafor’ (Schwarzer, Carpenter, & Rucker, 2015; Viechtbauer, 2010). If there were indications for publication bias, the trim-and-fill method (Duval, 2005; Duval & Tweedie, 2000a; 2000b) was used to calculate corrected ORs. In these cases, data from the trim-and-fill method are presented in the text along with the uncorrected estimates, while the presented figures contain the original uncorrected data only.

## Results

### Study selection

A total of 402 studies were identified in the initial search. One duplicate article was removed and 381 articles were removed after screening titles and abstracts. Seven articles were removed after a full-text review (see Fig. 1). A total of 13 publications, describing 12 unique cohorts, were included: see Table 1 and online Supplementary Table S1 for the details of each study.

**Table 1.** Characteristics of studies that investigated the relationship between LGB status and risk of PDs or PEs

Reference	Country	Study population	*N*^a^	Measure of sexual orientation	Measure of psychosis	OR (95% CI)^b^
**Psychotic disorder**						
Bolton and Sareen (2011)	USA	General population	33 958	Identification as L, G, or B	Self-reported history of diagnosis, made by professional, of schizophrenia or psychotic episode/illness	2.05 (1.48–2.84)^c^
Borgogna et al. (2022)	USA	Case-control study among students	477	Identification as L, G, or B	As above	6.39 (3.36–12.12)^c^
Chakraborty et al. (2011)	UK	General population	7403	Identification as not entirely heterosexual/ever had same-sex partner	Probable non-organic psychosis in year before interview^d^	3.09 (1.39–6.89)
Currier et al. (2015)	USA	Psychiatric Emergency Dept.	177	Identification as L, G, or B	PD	0.53 (0.07–4.30)^e^
Post (2011)	NLD	Case-control study in general population	1547	Identification as predominantly homosexual	Non-affective PD^f^	2.59 (1.28–5.25)^c^
Qi et al. (2020)	UK	General population	7403	As in Chakraborty et al. (2011)	Probable non-organic psychosis in year before interview ^d^	2.87 (1.09–7.53)
Skerrett et al. (2014)	AUS	Suicides	5996	Known in community as L, G, or B	PD	0.21 (0.03–1.70)^e^
**Psychotic experiences**						
Gevonden et al. (2014)	NLD	General population	5927	At least one same-sex partner in past year	Lifetime PE	3.25 (2.22–4.76)
Gevonden et al. (2014)	NLD	General population	5308	Equally, predominantly, or only attracted to own gender	Lifetime PE	2.28 (1.53–3.41)
Jacob et al. (2021)	UK	General population	7275	As in Chakraborty et al. (2011)	Past year PE	2.21 (1.64–2.99)^c^
Lu et al. (2023)	China	College students	4460	Identification as L, G, or B	Lifetime PE	1.60 (1.22–2.08)
Oh (2021)	USA	General population	5096	Any homosexual experience in past year	Lifetime PE	1.56 (1.01–2.41)
Oh et al. (2022)	USA	College students	109 975	Identification as L, G, or B	Past year PE	1.64 (1.56–1.73)
Pérez-Albéniz et al. (2023)	Spain	Adolescents	1790	Identification as L, G, or B	Lifetime PE	N.A.^g^

*Notes*: AUS, Australia; L, G, or B, lesbian, gay, or bisexual; N.A., not applicable; NLD, The Netherlands.

a Sample sizes per group are reported in online Supplementary Table S1.

b Covariate-unadjusted OR and 95% CI.

c Computed by the authors of this meta-analysis.

d According to International Classification of Diseases, 10th edition.

e OR computed by authors of this meta-analysis by changing the number of psychotic cases in the LGB group from 0 to 1.

f According to Diagnostic and Statistical Manual of Mental Disorders, 4th edition.

g Not applicable. The study did not use dichotomous measures.

All the studies were cross-sectional. Seven studies reported comparisons for PD (Bolton & Sareen, 2011; Borgogna, Aita, Trask, & Moncrief, 2022; Chakraborty, McManus, Brugha, Bebbington, & King, 2011; Currier et al., 2015; Post et al., 2021; Qi et al., 2020; Skerrett et al., 2014) and six assessed PE (Gevonden et al., 2014; Jacob et al., 2021; Lu et al., 2023; Oh, 2021; Oh et al., 2022; Pérez-Albéniz, Lucas-Molina, & Fonseca-Pedrero, 2023). The studies had been conducted in the USA (*n* = 5), the UK (*n* = 3), The Netherlands (*n* = 2), Spain (*n* = 1), China (*n* = 1), and Australia (*n* = 1), countries where homosexuality is legal.

Six studies collected their data from general population samples. The remaining studies included patients diagnosed with a PD (Post et al., 2021), college students (Borgogna et al., 2022; Lu et al., 2023; Oh et al., 2022), secondary school students aged 14–18 years (Pérez-Albéniz et al., 2023), individuals who underwent a standardized suicide risk assessment at a psychiatric emergency department (Currier et al., 2015), or individuals who died by suicide (Skerrett et al., 2014).

While all of the studies reported on sexual orientation, four studies also provided information on sexual activity, defined as ‘sexual intercourse’, ‘sexual experience’ (Chakraborty et al., 2011; Qi et al., 2020), ‘touching the genitals of another person in the preceding year’ (Gevonden et al., 2014), or ‘sexual experiences in the last 12 months’ (Oh, 2021). Questions about PEs concerned the past 12 months (Jacob et al., 2021; Oh et al., 2022) or the whole lifetime (Gevonden et al., 2014; Lu et al., 2023; Oh, 2021).

Since the corresponding author of the study conducted in Spain did not respond to our request for group sizes, ORs, or RRs, we could not include the results in our meta-analysis. The study found that adolescents with a homosexual or bisexual orientation reported significantly more symptoms (about 57% more) on the Prodromal Questionnaire-Brief (Loewy, Pearson, Vinogradov, Bearden, & Cannon, 2011) than their heterosexual peers.

Two studies derived different ORs of probable PD from the Adult Psychiatric Morbidity Survey in the UK (Chakraborty et al., 2011; Qi et al., 2020). Qi et al.'s (2020) investigation differed from that of Chakraborty et al. (2011) in that it excluded participants about whom information was obtained using proxy interviews and several individuals with missing data. Since it was not evident which result was more valid, we used the study that yielded the lowest OR (Qi et al., 2020).

### Quality assessment

The quality assessment results of our instrument were strongly correlated with the tool developed by the NIH (*r* = 0.68; *p* < 0.01; online Supplementary Fig. S1 and Tables S2 and S3), supporting the validity of our instrument. The results showed that one study, which examined the number of LGB people in Queensland, Australia, who had committed suicide, was of a distinctly lower quality (Skerrett et al., 2014). The researchers identified only 34 LGBs among 5999 suicide victims (0.57%) and no LGB individual with a diagnosis of a PD. As the authors acknowledged, the study was of limited quality, because the assignment of LGB status was often based on indirect indicators, such as being unmarried and childless, and the proportion of LGB individuals who died by suicide was low. Currier et al.'s (2015) study was of sufficient quality, but its goal was to explore whether patients at an emergency department would disclose their sexual orientation. It screened a small number of subjects (*n* = 177) and reported no PD among 21 subjects who identified themselves as LGB.

The quality of the remaining five studies on PD was high. The Adult Psychiatric Morbidity Survey in the UK (*n* = 7403) and the National Epidemiologic Survey on Alcohol and Related Conditions Wave 2 in the USA (*n* = 34 653) were large investigations of the general population (Bolton & Sareen, 2011; Chakraborty et al., 2011; Qi et al., 2020). Using a dataset of the Healthy Minds Study, Borgogna et al. (2022) asked 159 students who had reported to have been diagnosed with a PD for their sexual orientation; they then compared the results with those in a control group. A study from the Netherlands reported the proportion of individuals with LGB orientation in a large sample (*n* = 582) of patients diagnosed with a PD (Post et al., 2021).

Among the studies into PEs there was also one that was of lower quality (online Supplementary Fig. S1 and Tables S2 and S3). Lu et al.'s (2023) study was of questionable validity, because the proportion of college students who reported at least one PE was 60.1%. This figure is high if one considers that a systematic review found a median prevalence rate of self-reported PEs of 11.9% (Linscott & van Os, 2013). It is even higher if one takes into account that Lu et al. dropped 12 items from the analyses because they were very commonly reported. Another problem is that Lu et al. adjusted the results for the quality of relationships with parents, teachers and classmates, while a disturbance in these relationships may contribute to the increased ORs. All studies, except for the Spanish one, reached more than 100 LGB people. One study had a large sample of LGB college students, but the response rate was low (14%) (Oh et al., 2022).

### Meta-analyses

**Figure 2.**
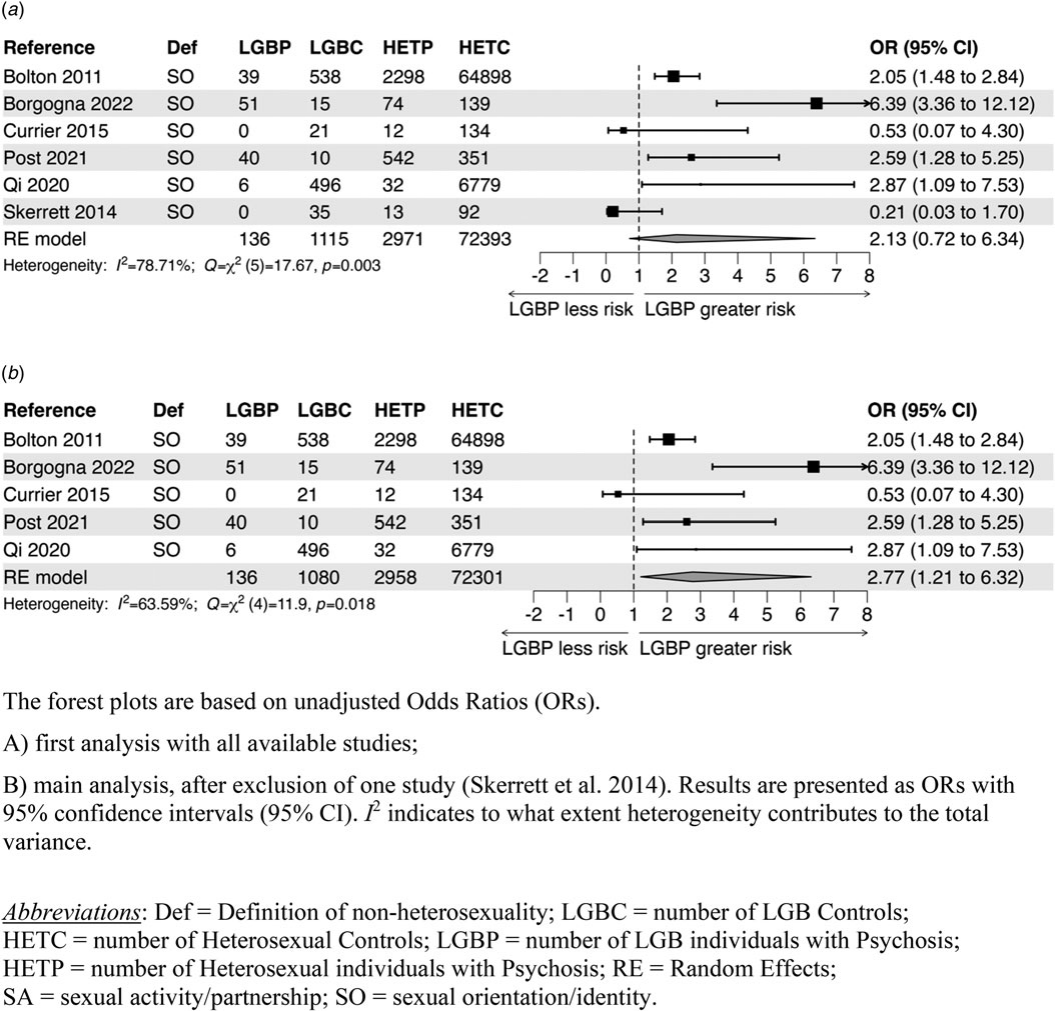
Meta-analysis of studies that examined the relationship between LGB status and risk for PDs.

The results of the heterogeneity tests were significant for the covariate-unadjusted ORs derived from all studies on PD, but not for the covariate-adjusted ORs (Fig. 2; online Supplementary Fig. S2A and Table S4). The heterogeneity for the covariate-unadjusted ORs remained significant after exclusion of Skerrett et al.'s paper. As for the studies on PEs, the heterogeneity test was significant for the unadjusted ORs, but not for the adjusted ones. Considering the pros and cons, we decided to use in each analysis an RE model, because the differences among studies were large.

Given the large variety of covariates for which the results were adjusted, in particular with reference to PD, to ensure comparability, the covariate-unadjusted pooled ORs are presented as the primary result, over the covariate-adjusted pooled ORs.

There was no indication of publication bias in the meta-analyses of covariate-unadjusted ORs from studies on PDs or PEs (online Supplementary Fig. S3 and Table S4). There was an indication (funnel plot asymmetry) of this type of bias in the meta-analysis of covariate-adjusted ORs from all studies on PDs (not after exclusion of Skerrett et al.'s paper), but not in the meta-analysis of all covariate-adjusted ORs for PEs. In cases of a significant bias test indicating funnel plot asymmetry, the trim-and-fill corrected effects (OR_tf_) are reported in the text.

#### Main findings for PDs

A first meta-analysis, based on all available studies, found that the unadjusted, pooled OR [95% CI] was not significantly increased for LGB people (pooled OR_RE_ = 2.13 [0.72–6.34]; *k* = 6) (see Fig. 2A). The covariate-adjusted, pooled OR (pooled OR_RE_ = 2.24 [0.90–5.58]; *k* = 5) was also not significantly increased and did not become significantly increased after adjustment for publication bias: pooled OR_RE-tf_ = 2.75 [0.94–3.99]; *k* = 5) (online Supplementary Fig. S2A and Table S4).

After excluding Skerrett et al.'s study, both the unadjusted, pooled OR (OR_RE_ = 2.77 [1.21–6.32]; *k* = 5) and the covariate-adjusted, pooled OR (OR_RE_ = 2.67 [1.53–4.66]; *k* = 4), were significantly increased (Fig. 2B; online Supplementary Fig. S2B and Table S4). Qi et al.'s (2020) study reported a lower OR when LGB status was based on any homosexual activity (OR = 1.55 [0.30–8.07]. When we used this value, the pooled OR remained significantly increased: unadjusted, pooled OR_RE_ = 2.56 [1.02–6.42]; adjusted, pooled OR_RE_ = 2.59 [1.37–4.89].

From two studies it was possible to derive separate effect sizes for males and females (Bolton & Sareen, 2011; Post et al., 2021). However, the 95% CI for the unadjusted, pooled OR_RE_ for LGB women was extremely wide (OR_RE_ = 2.23 [0.03–195.06]; *k* = 2) and, understandably, not significantly different from the corresponding figure for LGB men (unadjusted, pooled OR_RE_ for LGB men = 2.29 [0.54–9.78], *k* = 2; online Supplementary Table S4).

Two studies distinguished in their analyses between homosexual and bisexual individuals (Bolton & Sareen, 2011; Borgogna et al., 2022). The 95% CIs for the pertinent ORs were very wide and overlapping (online Supplementary Table S4).

#### Main findings for PEs

**Figure 3.**
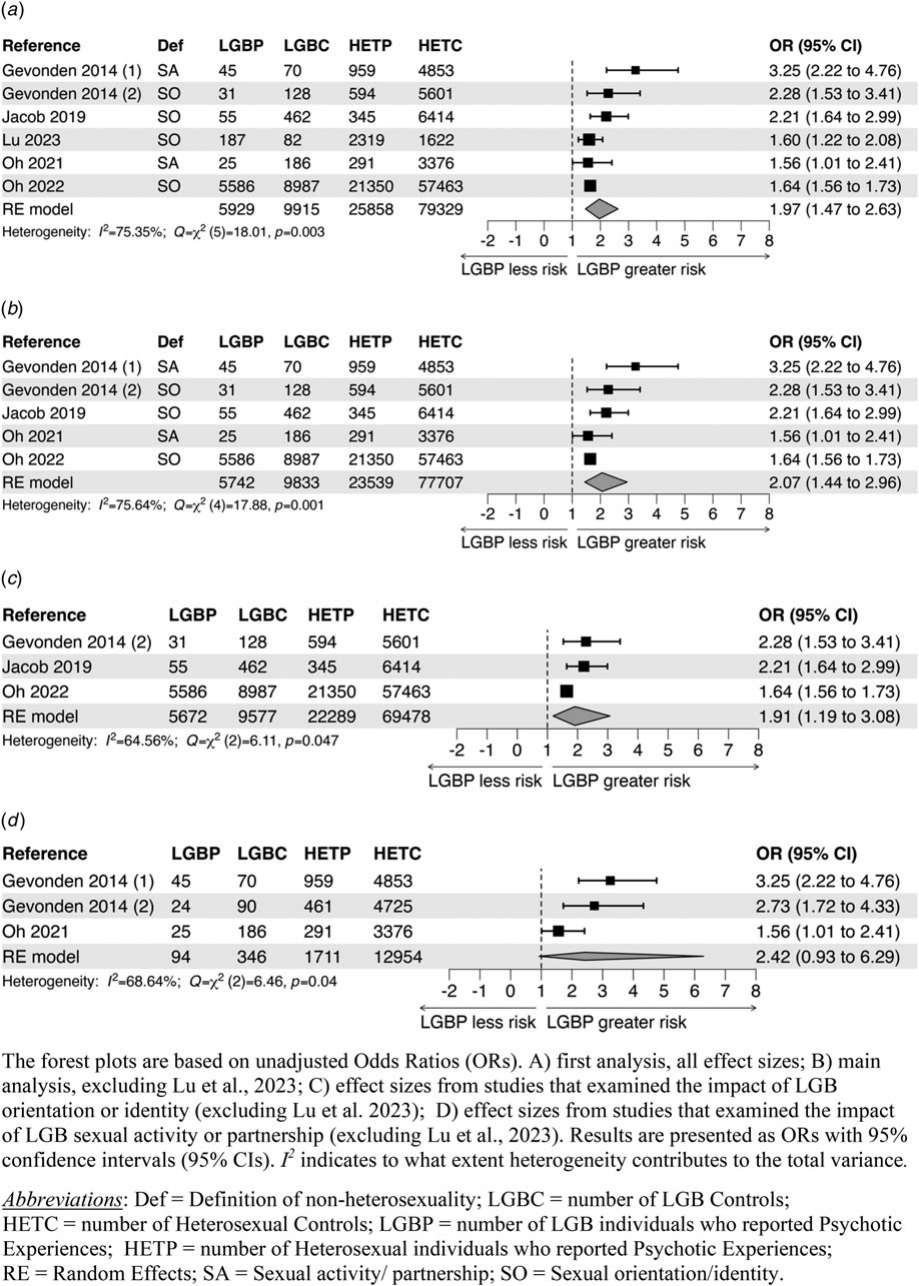
Meta-analysis of studies that examined the relationship between LGB status and risk for PEs.

The meta-analysis revealed that LGB people reported more PEs than heterosexuals. Both the unadjusted, pooled OR (OR_RE_ = 1.97 [1.47–2.63]; *k* = 6) and the covariate-adjusted, pooled OR (OR_RE_ = 1.85 [1.50–2.28]; *k* = 6; were significantly increased (Fig. 3A; online Supplementary Fig. S4A and Table S4). After exclusion of the study by Lu et al. (2023), the unadjusted, pooled OR_RE_ (2.07 [1.44–2.96]; *k* = 5) and the covariate-adjusted pooled OR_RE_ (1.85 [1.50–2.28]; *k* = 5) became slightly higher (Fig. 3B).

The OR for individuals who reported some kind of homosexual or bisexual activity was more increased (unadjusted, pooled OR_RE_ = 2.42 [0.93–6.29]; *k* = 3) than the OR for subjects who reported only a homosexual or bisexual orientation (unadjusted pooled OR_RE_ = 1.91 [1.19–3.08]; *k* = 3) (Figs 3C and 3D; online Supplementary Figs S4C and S4D and Table S3).

Two papers reported separate estimates for individuals with a homosexual or bisexual orientation (Lu et al., 2023; Oh et al., 2022). In both studies the risk for bisexuals was somewhat higher than that for homosexuals. The results of a meta-analysis showed that the OR for bisexuals was slightly more increased (unadjusted, pooled OR_RE_ = 1.73 [1.68–1.78]; *k* = 2) than the OR for homosexuals (unadjusted pooled OR_RE_ = 1.35 [1.07–1.69]; *k* = 2), although CIs just overlapped (online Supplementary Table S3).

Only one study reported separate effect sizes for males and females; the 95% CIs were overlapping (Lu et al., 2023).

#### Mediators

One study conducted a mediation analysis on the association between LGB status and risk of PD (Post et al., 2021) and three studies did this for PEs (Gevonden et al., 2014; Jacob et al., 2021; Pérez-Albéniz et al., 2023). They reported significant effects of several variables, including bullying victimization (three studies), discrimination (two studies), and childhood trauma (two studies). Interestingly, two studies of PEs examined the mediating role of drug abuse and reported that the contribution of this variable was small compared to that of psychosocial stressors (online Supplementary Table S5). Finally, the researchers in Spain reported a significant negative interaction between LGB orientation and parental support for PEs (Pérez-Albéniz et al., 2023). In other words, the effect of sexual orientation on reporting of PEs was moderated to an important and significant degree by this type of support. The finding suggests a protective effect.

#### Limitations of the relevant studies

An important goal of a systematic review is to address shortcomings in the literature. We noted several limitations. First, there were only cross-sectional studies and no longitudinal investigations. However, given the large numbers required for a prospective, longitudinal study, such a study seems hardly feasible. Second, most studies were based on the willingness of individuals to report PEs or to report that they had been given a diagnosis of PD by a health professional. A notable exception is the investigation by Post et al., (2021), which was based on a large sample of patients that had already been diagnosed with a (non-affective) PD. Third, of the studies on PD only one distinguished between affective and non-affective PD (Post et al., 2021). Thus, it is not possible to conclude about any difference in the risk for affective and non-affective PD. Fourth, some studies identified substance use as a potential mediator and collecting data on substance use is subject to reporting biases (Gevonden et al., 2014; Jacob et al., 2021). Finally, and there is probably no solution to this problem, measuring LGB status was based on self-identification, which may have resulted in social desirability bias due to the stigma of belonging to a sexual minority group.

## Discussion

This systematic review retrieved five studies of good quality that compared the risk of PDs for LGB individuals to that for heterosexual individuals and five studies of good quality that did the same regarding PEs. The odds of PD for LGB individuals were significantly increased and about twice that for heterosexual individuals. Their odds of PEs were also significantly increased, by about the same extent.

To our knowledge this is the first systematic review and meta-analysis that compared the risk of psychotic phenomena for the LGB population to that for the heterosexual population. A strength of this paper is the comprehensive literature survey and the critical assessment of the quality of the studies.

### Interpretation

There are many indications that LGB people are more likely to experience stigma, prejudice, and discrimination. The minority stress model posits that this leads to a higher risk to develop common mental disorders, like depression and anxiety (Meyer, 2003), but does not mention a higher risk of non-affective PD. For this reason, the social defeat hypothesis complements the minority stress theory and adds greater specificity by positing that low status and repeated humiliation increase the risk for schizophrenia and other non-affective PDs. This line of thought is further supported by recent findings of increased risks of such disorders for persons with gender dysphoria (e.g. Termorshuizen et al., 2023).

There are several factors consistent with minority stress theory and the social defeat hypothesis that can be explored in future studies to explain the higher risk of LGB individuals relative to heterosexual individuals. Homophobia and heterosexism are strong drivers of health disparities, resulting in higher exposures to stressors (e.g. discrimination, hate crimes, trauma, adverse childhood experiences) and, in many countries, lower access to health promoting resources (e.g. housing, health care). For some LGB individuals, experiences of discrimination may be linked to high-risk behaviors, such as illicit drug use (Caceres et al., 2017; McCabe, Bostwick, Hughes, West, & Boyd, 2010), which can directly impact or combine with other high-risk behaviors (e.g. chemsex behavior; Maxwell, Shahmanesh, & Gafos, 2019) to increase risk for psychosis.

Another approach for further study is a search for protective and resilience factors. Pérez-Albéniz et al. (2023) found that adolescents with homosexual or bisexual orientation who feel supported by their parents reported fewer PEs. This finding is critical and deserves further research.

### Limitations

One limitation is that we excluded papers that reported figures for LGBTQ+ individuals more broadly, which included many LGB individuals. It is unlikely, however, that our more stringent exclusion criteria would have significantly influenced our results, since most of these studies on LGBTQ+ broadly defined reported increased risk for sexual minority groups (e.g. Barr, Bigdeli, & Meyers, 2022; Savill, Nguyen, Shim, & Loewy, 2022). Future research can explore queer orientation/identity with greater nuance and specificity. Second, despite our efforts to include all relevant papers, we may have missed some studies, which is always a possibility when conducting systematic searches. Finally, it is important to note that the terms ‘lesbian’, ‘gay’, and ‘bisexual’ do not adequately capture the complexity of human sexuality, and future research can unpack these terms, and explore the various facets of sexual identity, desire, and behavior more closely.

## Conclusion

The results suggest that the risk of developing psychotic symptoms or PDs is increased for LGB individuals compared to heterosexual individuals and underline the importance of combating discrimination. Future studies can further explore the risk and protective factors, while formulating efforts to reduce this disparity.

## Supporting information

Selten et al. supplementary material 1 Selten et al. supplementary material

Selten et al. supplementary material 2 Selten et al. supplementary material

Selten et al. supplementary material 3 Selten et al. supplementary material

Selten et al. supplementary material 4 Selten et al. supplementary material

## Supplementary material

The supplementary material for this article can be found at https://doi.org/10.1017/S0033291724002253.
