# Supplementary material for: Psychosis risk for lesbian, gay, and bisexual individuals: systematic review and meta-analysis
Source: Psychol Med. 2024 Sep 25;54(13):3581–90. doi: 10.1017/S0033291724002253 (PMC11536103; doi:10.1017/S0033291724002253)
Supplement: Selten et al. supplementary material 1 — Selten et al. supplementary material [file S0033291724002253sup001.pdf]

RE: Odds Ratio in jullie artikel over Sexual minority status

Veling, WA (psy) <w.veling@umcg.nl>
Di 14-11-2023 11:59
Aan:Selten, Jean-Paul (NP) <jp.selten@maastrichtuniversity.nl>

Dear Jean-Paul,

You are right.
Here the Odds Ratio calculation for LGB status and risk for psychosis in the GROUP data, cases compared to controls:

Risk Estimate

	Value	95% Confidence Interval	
		Lower	Upper
Odds Ratio for status participant (control / patient)	2,590	1,279	5,247
For cohort LBG = no	1,044	1,015	1,074
For cohort LBG = yes	,403	,204	,796
N of Valid Cases	943		

Adjusted for age and gender:

Variables in the Equation

		B	S.E.	Wald	df	Sig.	Exp(B)
Step 1[a]	LBG	,907	,382	5,636	1	,018	2,478
	Age	-,054	,008	44,841	1	<,001	,948
	Gender	-1,254	,148	72,273	1	<,001	,285
	Constant	4,113	,365	127,115	1	<,001	61,114

a. Variable(s) entered on step 1: LBG, Age, Gender.

Here the Odds Ratio calculation for LGB status and risk for psychosis in the GROUP data, cases compared to controls+siblings:

Variables in the Equation

		B	S.E.	Wald	df	Sig.	Exp(B)
Step 1[a]	LBG	,645	,236	7,476	1	,006	1,907
	Constant	-,540	,054	99,823	1	<,001	,583

a. Variable(s) entered on step 1: LBG.

Adjusted for age and gender:

Variables in the Equation

		B	S.E.	Wald	df	Sig.	Exp(B)
Step 1[a]	LBG	,570	,248	5,273	1	,022	1,768
	Age	-,027	,007	17,301	1	<,001	,973
	Gender	-1,275	,116	119,811	1	<,001	,279
	Constant	2,191	,282	60,273	1	<,001	8,945

a. Variable(s) entered on step 1: LBG, Age, Gender.
