# Supplementary material for: Psychosis risk for lesbian, gay, and bisexual individuals: systematic review and meta-analysis
Source: Psychol Med. 2024 Sep 25;54(13):3581–90. doi: 10.1017/S0033291724002253 (PMC11536103; doi:10.1017/S0033291724002253)
Supplement: Selten et al. supplementary material 3 — Selten et al. supplementary material [file S0033291724002253sup003.docx]

**Supplementary Materials for:**

**Psychosis Risk for Lesbians, Gays and Bisexuals:**

**Systematic Review and Meta-analysis**

Jean-Paul Selten, Hussam Alrashed, Hans Oh, Gabriëlla A. M. Blokland

**Table of Contents**

[Supplementary Methods 2](#_Toc165500208)

[Search Strategy 2](#_Toc165500209)

[Instruments to measure quality of studies 4](#_Toc165500210)

[Supplementary Figures](#_Toc165500211) 4

[Supplementary Figure 1. Quality scores based on (A) tool developed by the authors of this study and (B) tool for observational cohort and cross-sectional studies, developed by National Institutes of Health, versus effect size. 6](#_Toc165500213)

[Supplementary Figure 2. Meta-analysis forest plots for psychotic disorder; covariate-adjusted ORs. 7](#_Toc165500214)

Supplementary Figure 3. Funnel plots………………………………………………………8

[Supplementary Figure 4. Meta-analysis forest plots for psychotic experiences; covariate-adjusted ORs. 9](#_Toc165500215)

[Supplementary Tables](#_Toc165500216) 10

Supplementary Table 1. Studies that examined the relationship between LGB status

and psychosis risk: diagnostic criteria or interviews used, numbers of participants, by sexual orientation and presence/absence of psychotic features; and covariates 10

[Supplementary Table 2. Quality of studies on relationship between LGB-status and risk of psychotic disorder or psychotic experiences, measured using the quality assessment tool for observational cohort and cross-sectional studies^1^ (National Institute of Health). 11](#_Toc165500217)

[Supplementary Table 3. Quality of studies on relationship between LGB-status and risk of psychotic disorder or psychotic experiences, measured using instrument developed by authors. 12](#_Toc165500218)

[Supplementary Table 4. Statistics from all meta-analyses on the relationship between LGB-status and risk of psychotic disorder or psychotic experiences. 13](#_Toc165500219)

[Supplementary Table 5. Possible mediators of an association between LGB status and psychotic phenomena and the proportions of the effect mediated. 14](#_Toc165500220)

[References 15](#_Toc165500221)

Supplementary Methods

## Search Strategy

The protocol of the study was registered on the International Prospective Register of Systematic Reviews (PROSPERO; https://www.crd.york.ac.uk/prospero/) under number: CRD42022354853. On January 29^th^, 2024 we conducted a final search in PubMed, Embase, Web of Science and PsycINFO, using the following search terms.

For PubMed: ("Schizophrenia Spectrum and Other Psychotic Disorders"[MeSH Terms] OR "psychotic"[Title/Abstract] OR "psychosis"[Title/Abstract] OR "schizo*"[Title/Abstract]) AND ("homosexuality"[MeSH Terms] OR "bisexuality"[MeSH Terms] OR "lesbian*"[Title/Abstract] OR "gay"[Title/Abstract] OR "gays"[Title/Abstract] OR "homosexual*"[Title/Abstract] OR "bisexual*"[Title/Abstract] OR "same sex"[Title/Abstract] OR "sexual minorit*"[Title/Abstract] OR "sexual orientation*"[Title/Abstract] OR "non-heterosexual*"[Title/Abstract] OR "LGB"[Title/Abstract] OR "MSM"[Title/Abstract] OR "lgbt*"[Title/Abstract]) AND ("prevalence"[MeSH Terms] OR "risk factors"[MeSH Terms] OR "epidemiology"[MeSH Subheading] OR "incidence"[MeSH Terms] OR "risk"[Title/Abstract] OR "incidence"[Title/Abstract] OR "prevalen*"[Title/Abstract] OR "odds"[Title/Abstract] OR "ratio"[Title/Abstract])

For PsycINFO: (DE "Psychosis" OR DE "Schizophrenia" OR TI ( "psychotic" OR "psychosis" OR "schizo*" ) OR AB ( "psychotic" OR "psychosis" OR "schizo*" ) OR KW ( "psychotic" OR "psychosis" OR "schizo*" ) ) AND (DE "Homosexuality" OR DE "Lesbianism" OR DE "Male Homosexuality" OR DE "Bisexuality" OR TI ( "lesbian*" OR "gay" OR "gays" OR "homosexual*" OR "bisexual*" OR "same sex" OR "sexual minorit*" OR "sexual orientation*" OR "non-heterosexual*" OR "LGB" OR "MSM" OR "lgbt*" ) OR AB ( "lesbian*" OR "gay" OR "gays" OR "homosexual*" OR "bisexual*" OR "same sex" OR "sexual minorit*" OR "sexual orientation*" OR "non-heterosexual*" OR "LGB" OR "MSM" OR "lgbt*" ) OR KW ( "lesbian*" OR "gay" OR "gays" OR "homosexual*" OR "bisexual*" OR "same sex" OR "sexual minorit*" OR "sexual orientation*" OR "non-heterosexual*" OR "LGB" OR "MSM" OR "lgbt*" ) ) AND (DE "Risk Factors" OR DE "Epidemiology" OR TI ( "risk" OR "incidence" OR "prevalen*" OR "odds" OR "ratio" ) OR AB ( "risk" OR "incidence" OR "prevalen*" OR "odds" OR "ratio" ) OR KW ( "risk" OR "incidence" OR "prevalen*" OR "odds" OR "ratio" ) )

For Embase: ((exp psychosis/ or ("psychotic" or "psychosis" or "schizo*").ti,ab,kf) and (exp homosexuality/ or exp bisexuality/ or ("lesbian*" or "gay" or "gays" or "homosexual*" or "bisexual*" or "same sex" or "sexual minorit*" or "sexual orientation*" or "non-heterosexual*" or "LGB" or "MSM" or "lgbt*").ti,ab,kf) and (exp prevalence/ or exp risk factor/ or exp incidence/ or epidemiology.fx or ("risk" or "incidence" or "prevalen*" or "odds" or "ratio").ti,ab,kf)) not ("conference abstract" or preprint or editorial or letter or note).pt

For Web of Science: (TS=("psychotic" OR "psychosis" OR "schizo*" )) AND (TS=("lesbian*" OR "gay" OR "gays" OR "homosexual*" OR "bisexual*" OR "same sex" OR "sexual minorit*" OR "sexual orientation*" OR "non-heterosexual*" OR "LGB" OR "MSM" OR "lgbt*" )) AND (TS=("risk" OR "incidence" OR "prevalen*" OR "odds" OR "ratio"))

Abstracts and unpublished papers were excluded. We checked by hand the reference lists of the twelve included papers for more publications. The search was conducted with the help of a librarian.

**Justification of exclusion of 7 papers after assessment for eligibility**

The studies by Moore and Selzer (1963) and Planansky and Johnston (1962) did not include a heterosexual control group. The papers by Selten (2012) and Oh (2024) were doublures with other papers by the same authors. The outcomes of the studies by Biernbaum and Ruscio (2004) and Blashill (2021) were not psychotic disorder or psychotic experience. The paper by Savill, Nguyen, Shim and Loewy (2022) reported results for LGBs, transgenders and queers.

**References:**

Biernbaum, M.A, Ruscio, M. (2004). Differences between matched heterosexual and non-heterosexual college students on defense mechanisms and psychopathological symptoms. *J Homosex, 48*(1):125-41.

Blashill, A.J., Fox, K., Feinstein, B.A., Albright, C.A., Calzo, J.P. (2021). Nonsuicidal self-injury, suicide ideation, and suicide attempts among sexual minority children. *J Consult Clin Psychol,* *89*(2):73-80.

Moore, R.A., Selzer, M.L. (1963). Male homosexuality, paranoia and the schizophrenias. *Am J Psychiatry,* *119*(8): 743-747.

Oh, H.Y., Jacob, L., Smith, L., Leaune, E., Zhou, S., Shin, J.I., Koyanagi, A. (2024). Sexual Minority Status and Psychotic Experiences Among Young Adult College Students in the United States*. J Homosex*, *71*(4): 916-933.

Planansky, K., Johnstone, R. (1963). The incidence and relationship of homosexual and paranoid features in schizophrenia. *J Mental Sci,* *108*(whole no 456): 604-615.

Savill, M., Nguyen, T., Shim, R.S., Loewy, R.L. (2022). Online Psychosis Screening: Characterizing an Underexamined Population to Improve Access and Equity. *Psychiatr Serv*, *73*(9):1005-1012.

Selten, J.P. (2012). Testing the social defeat hypothesis in another minority: are psychotic symptoms more prevalent in a non-heterosexual population? *Schizophr Res*. *136*, 50.

## Instruments to measure quality of studies

We developed instruments to measure the quality of studies that compared risks of psychotic disorder or psychotic experiences for LGB-people to that for heterosexual people (below). We assigned more points to studies of higher quality.

| **A. Psychotic Disorder Points** |
| --- |
| **1. Validity of context** |
| Community survey 2 |
| Treatment setting / Hospital / Case Register 1 |
| Other/ Not specified 0 |
| **2. Sample size** |
| Number of LGBs with psychotic disorder is 20 or more 2 |
| Number of LGBs with psychotic disorder is less than 20 0 |
| **3. Assessment sexual orientation** |
| Interview/questionnaire on sexual orientation/activity, more than 1 item 2 |
| Interview/questionnaire with 1 item 1 |
| No interview or questionnaire 0 |
| **4. Assessment psychotic disorder** |
| Semi-structured diagnostic interview 2 |
| Structured diagnostic interview/ clinical diagnosis/ case notes 1 |
| Unspecified 0 |
| **5. Classification psychotic disorder** |
| Classification/Diagnostic system reported 2 |
| Own system Or symptoms described 1 |
| No system/ not described 0 |
| **6. Interrater reliability for psychotic disorder** |
| Reported 1 |
| Not reported 0 |
| **7.Statistical analysis** |
| Reports 95% Confidence Interval for odds ratio (OR) or relative risk (RR)  (LGBs vs heterosexuals) 2 |
| No 95% CI 0 |
| Adjustment of OR or RR for age and sex 1 |
| No adjustment 0 |
| Reports results for homosexuals and bisexuals 1 |
| Not reported 0 |
| Reports results for males and females 1 |
| Not reported 0 |
| **Maximum score = 16 points** |

| **B. Psychotic Experiences**  **Points** |
| --- |
| **1. Validity of context.** |
| General population survey that concerns both sexes and wide age range 2 |
| Section or subgroup of general population (e.g. certain age-group or sex) 1 |
| Other/Not specified |
| **2. Response rate** |
| > 50% 1 |
| < 50% 0 |
| **3.Sample size** |
| Number of LGBs: > 1000 3 |
| Number of LGBs: 500 or > 500 2 |
| Number of LGBs: 100 or > 100 1 |
| Number of LGBs: < 100 0 |
| **4.Assessment sexual orientation** |
| Interview/questionnaire on sexual orientation/activity, more than 1 item 2 |
| Interview/questionnaire with 1 item 1 |
| No interview or questionnaire 0 |
| **5.Questionnaire for psychotic experiences** |
| Asks questions about at least 10 different psychotic experiences 1 |
| Less than 10 psychotic experiences 0 |
| Reports time frame (e.g. past year, lifetime) 1 |
| Does not report time frame 0 |
| **6.Statistical analysis** |
| Reports 95% Confidence Interval for odds ratio (OR) or relative risk (RR)  (LGBs vs heterosexuals) 2 |
| No 95% CI 0 |
| Adjustment of OR or RR for age and sex 1 |
| No adjustment 0 |
| Reports results for homosexuals and bisexuals 1 |
| Not reported 0 |
| Reports results for males and females 1 |
| Not reported 0 |
| **Maximum score = 15 points.** |

|  | 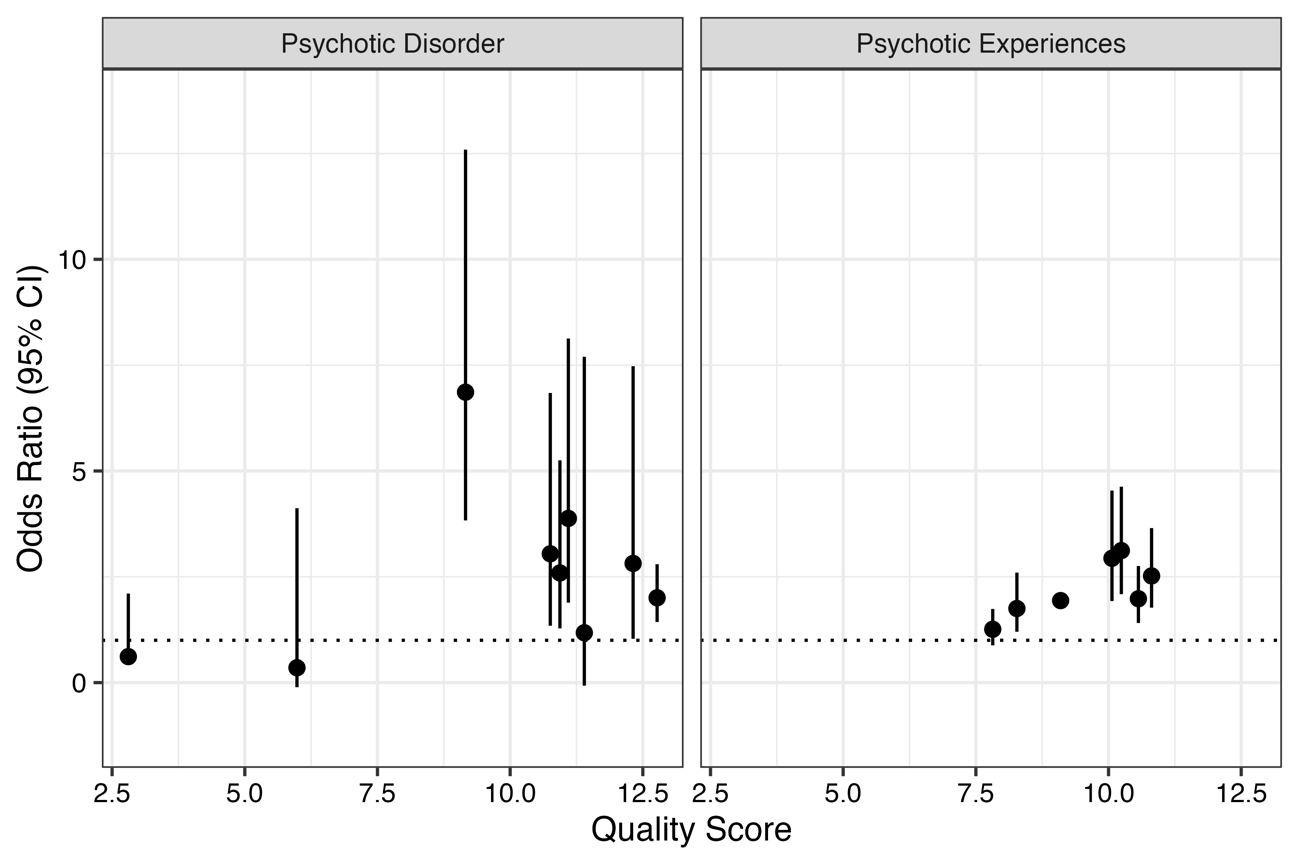 |
| --- | --- |
|  | 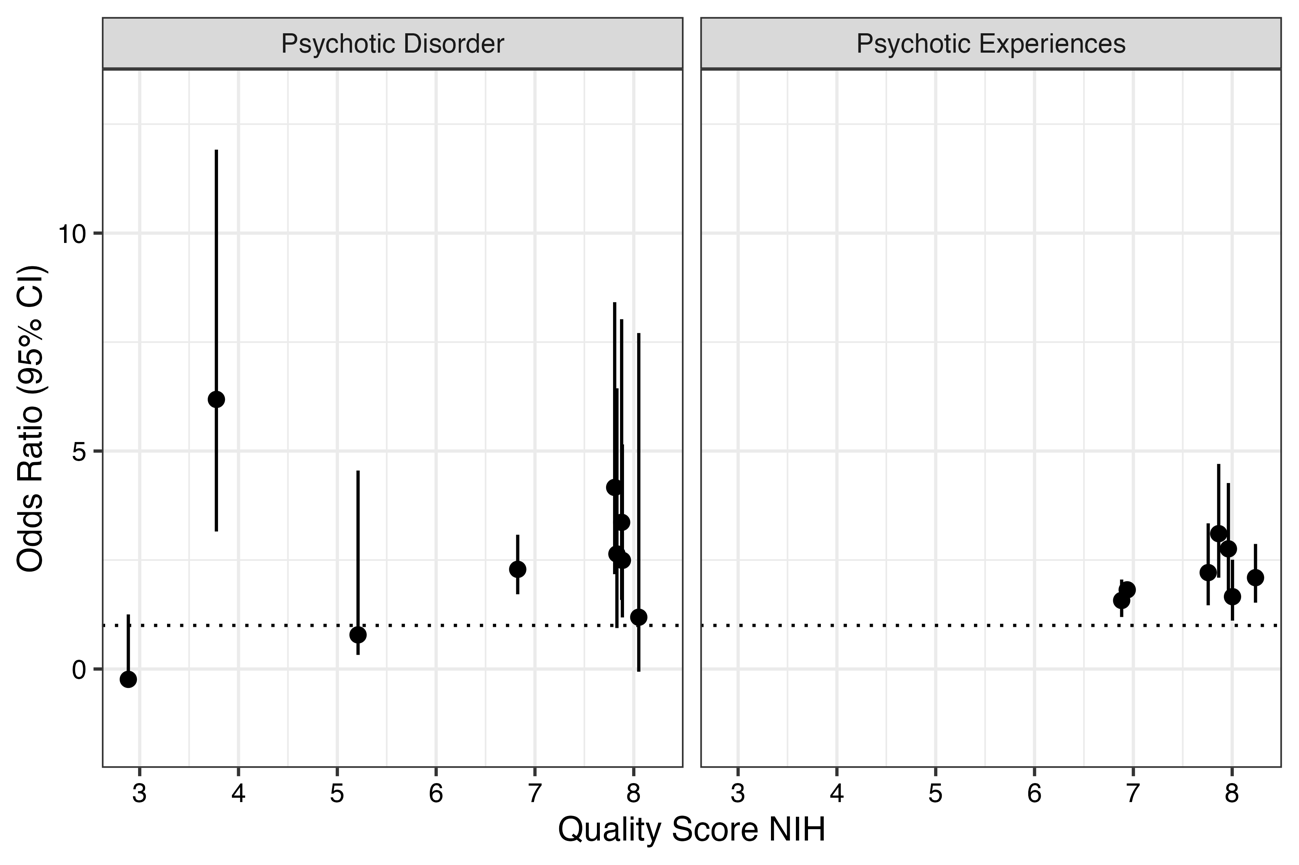 |

## Supplementary Figure 1. Quality scores based on (A) tool developed by the authors of this study and (B) tool for observational cohort and cross-sectional studies, developed by National Institutes of Health, versus effect size.

Note: Odds ratios ± 95% confidence interval (CI) displayed in the whisker plot are the covariate-unadjusted estimates.

## Supplementary Figure 2. Meta-analysis forest plots for psychotic disorder; covariate-adjusted ORs.

A.


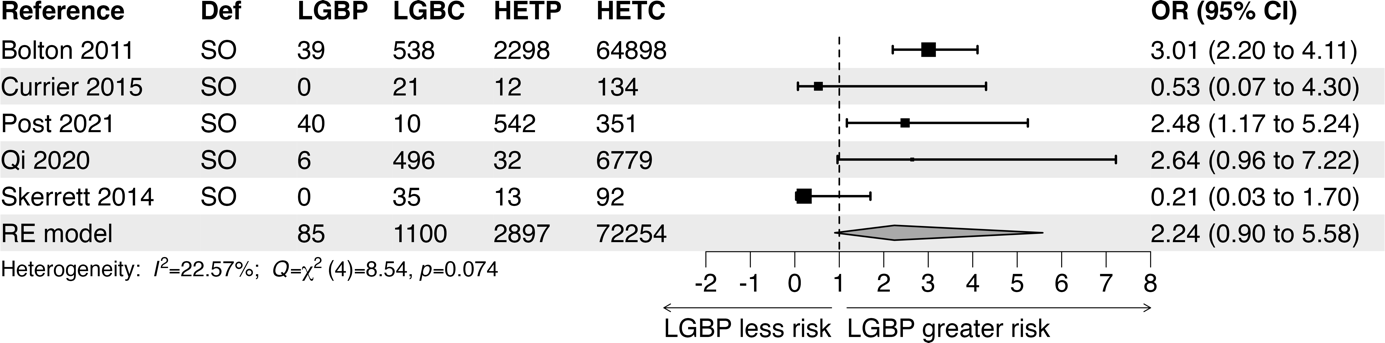


B.


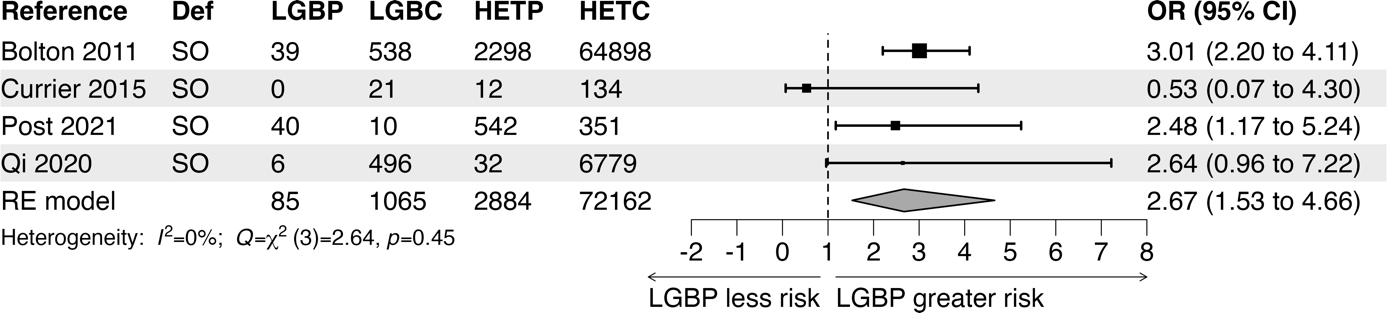


Meta-analysis of studies that examined the relationship between LGB status and risk for psychotic disorder. Forest plots are based on covariate-adjusted Odds Ratios (ORs). A) first analysis with all available studies; B) main analysis, after exclusion of one study. Results are presented as Odds Ratios (ORs) with 95% confidence intervals (95% CIs). ORs have been adjusted for the variables listed in Table 1. *I*^2^ indicates to what extent heterogeneity contributes to the total variance.

*Abbreviations*: Def = Definition of non-heterosexuality; LGBC = number of LGB controls; HETC = number of Heterosexual Controls; LGBP = number of LGB individuals with Psychosis; HETP = number of Heterosexual Individuals with Psychosis; RE = Random Effects; SO = sexual orientation/identity.

Supplementary Figure 3. Funnel plots for studies that examined the relationship between LGB status and psychosis risk.

| A. Psychotic Disorder |  |
| --- | --- |
| 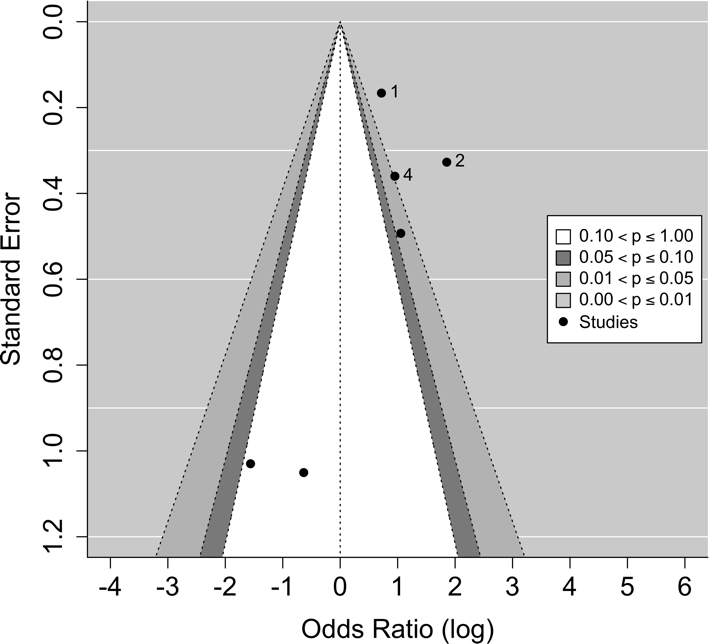 |  |
| B. Psychotic Experiences |  |
| 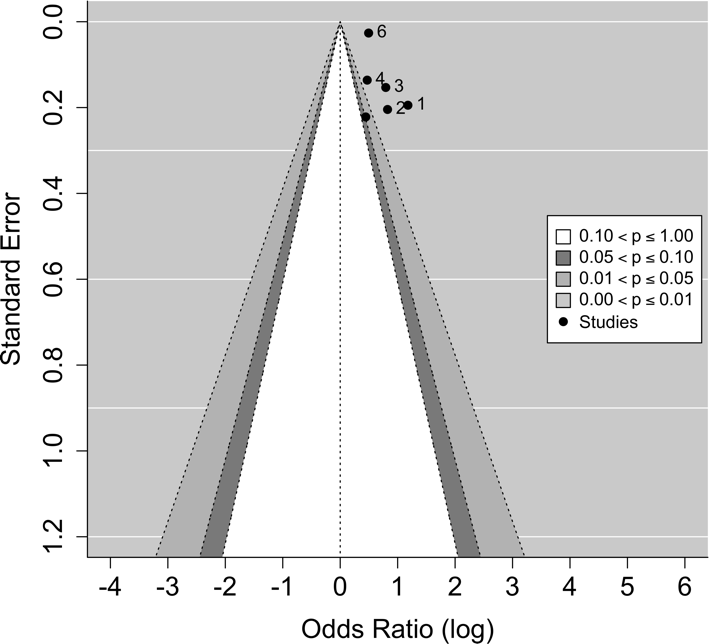 |  |

Note: The funnel plots are based on unadjusted odds ratios (ORs). The outer dashed lines indicate the triangular region within which 95% of studies are expected to lie in the absence of both biases and heterogeneity. Note that the funnel is centered at 0, the log OR which corresponds to an OR of 1, i.e., the value under the null hypothesis of no effect. The white region in the middle corresponds to p-values greater than .1, the region outside of the funnel corresponds to p-values below .01.

## Supplementary Figure 4. Meta-analysis forest plots for psychotic experiences; covariate-adjusted ORs.

A.


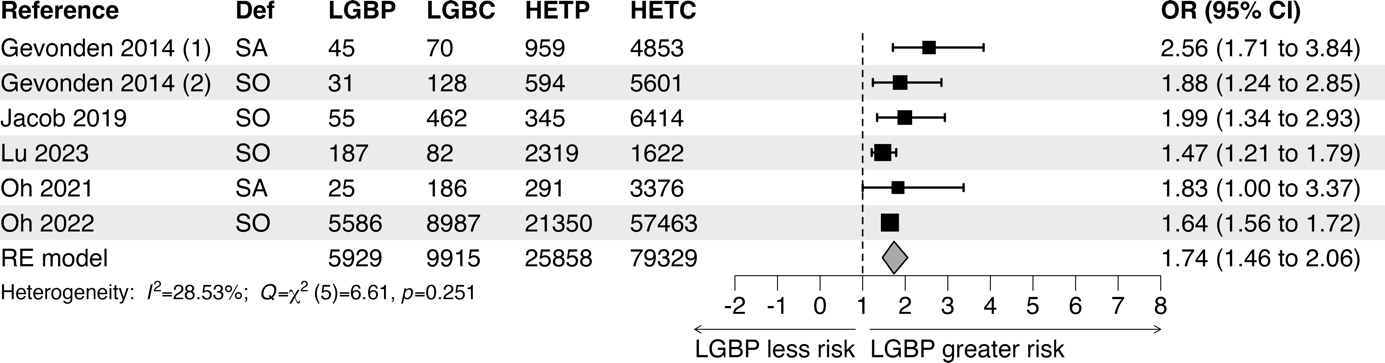


B.


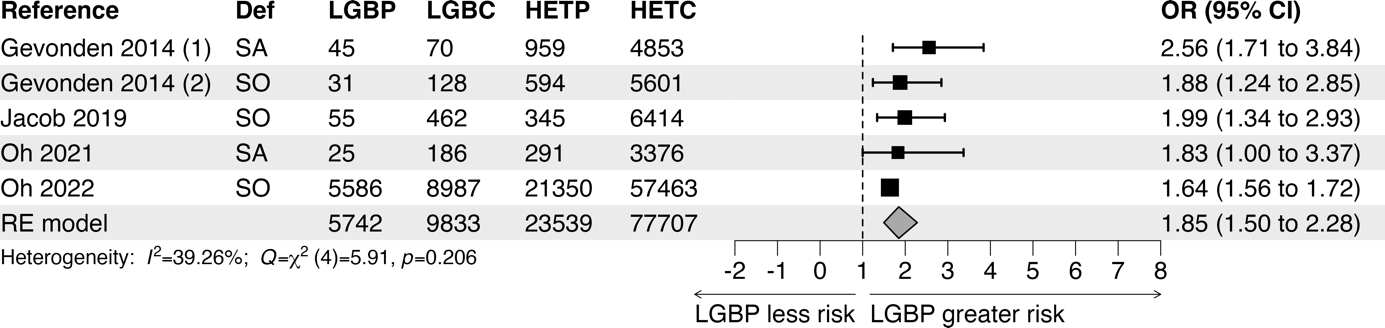


C.


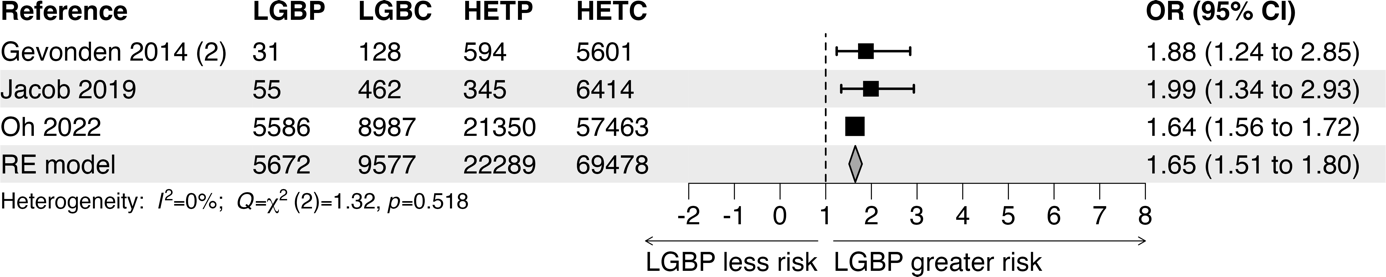


D.


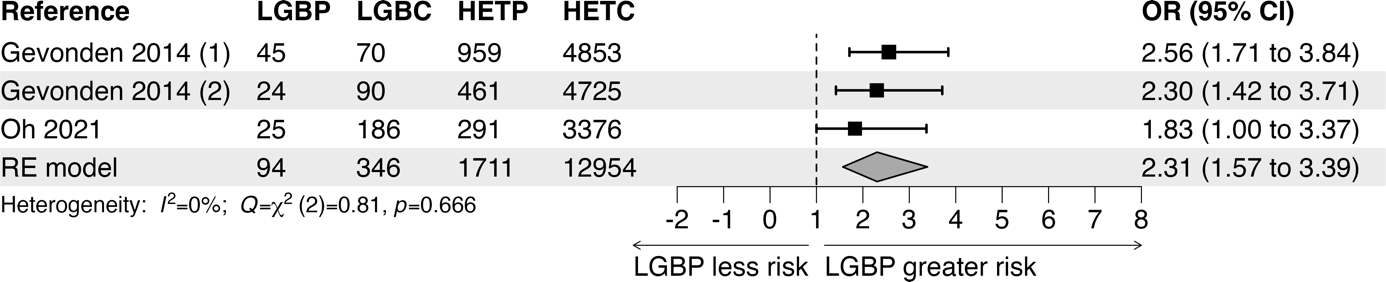


Meta-analysis of studies that examined the relationship between LGB status and risk for psychotic experiences, by type of LGB status. Forest plots are based on covariate-adjusted ORs. A) primary analysis, all effect sizes; B) main analysis, after exclusion of one study (Lu et al., 2023); C) effect sizes from studies that examined the impact of LGB orientation or identity (after exclusion of Lu et al., 2023); D) effect sizes from studies that examined the impact of LGB sexual activity or partnership (after exclusion of Lu et al., 2023). Results are presented as Odds Ratios (ORs) with 95% confidence intervals (95% CIs). ORs have been adjusted for the variables listed in Table 1. *I^2^* indicates to wat extent heterogeneity contributes to the total variance.

*Abbreviations*: Def = Definition of non-heterosexuality; FE = Fixed Effects; LGBC = number of LGB Controls; HETC = number of Heterosexual Controls; LGBP = number of LGB individuals with psychotic experiences HETP = number of Heterosexual individuals with psychotic experiences; RE = Random Effects; SA = sexual activity/partnership; SO = sexual orientation/identity.

**Supplementary Table 1. Studies that examined the relationship between LGB status and psychosis risk: diagnostic criteria**

**or interviews used, numbers of participants by sexual orientation and presence/absence of psychotic features, and covariates.**

| **Reference** | **Diagnostic Criteria or**  **Diagnostic Interview** | **Numbers of Heterosexuals without features of psychosis** | **Numbers of Heterosexuals with features of psychosis** | **Numbers of**  **LGBs without features of psychosis** | **Numbers of LGBs with features of psychosis** | **Covariates** |
| --- | --- | --- | --- | --- | --- | --- |
|  |  |  |  |  |  |  |
| ***Psychotic Disorder*** |  |  |  |  |  |  |
| Bolton 2011 | Self-report | 32,359 | 1,149 | 538 | 39 | a |
| Borgogna 2022 | Self-report | 139 | 74 | 15 | 51 | b |
| Chakraborty 2011 | ICD-10 | 6,768 | 26 | 659 | 8 | c |
| Currier 2015 | DSM-IV | 134 | 12 | 21 | 0 | d |
| Post 2011 | DSM-IV | 196 | 134 | 4 | 11 | e |
| Qi 2020 | ICD-10 | 6,761 | 33 | 350 | 2 | f |
| Skerrett 2014 | Not specified | 92 | 13 | 35 | 0 | g |
| ***Psychotic***  ***Experiences*** |  |  |  |  |  |  |
| Gevonden 2014 | CIDI 1.1 | 4,853 | 959 | 70 | 45 | h |
| Gevonden 2014 | CIDI 3.0 | 4,725 | 461 | 90 | 24 | i |
| Jacob 2021 | PSQ | 6,504 | 371 | 6,504 | 771 | j |
| Lu 2023 | CAPE | 1,625 | 2,319 | 82 | 187 | k |
| Oh 2021 | CIDI 3.0 | 3,376 | 291 | 186 | 25 | l |
| Oh 2022 | CIDI 3.0 | 57,463 | 21,350 | 8,987 | 5,586 | m |
| Pérez-Albéniz 2023 | PQ-B, no dichotomous  Psychosis measure |  |  |  |  | n |

**Notes:** *Diagnostic criteria/interview:* CAPE = Community Assessment of Psychic Experiences; CIDI = Composite International Diagnostic Interview;

DSM-IV = Diagnostic and Statistical Manual of Mental Disorders, Fourth Edition; ICD-10 = International Classification of Diseases 10^th^ Revision;

PSQ = Psychosis Screening Questionnaire. PQ-B= Prodromal Questionnaire-Brief.

*Covariates:* a) age, education level, IQ, SES, marital status, urban upbringing; b) none; c) none; d) none; e) age, sex; f) age, sex, education level; g) none;

h) age, sex, education level, cannabis use, illicit drug use, marital status, migration, urban upbringing; i) age, sex, education level, cannabis use, illicit drug use,

marital status, migration, urban upbringing; j) age, sex, ethnicity; k) grade, race, family location, financial situation, relationship with parents, with teachers,

with classmates; l) age, sex, education level, SES; m) age, sex, ethnicity, survey timing n) age.

## Supplementary Table 2. Quality of studies on relationship between LGB-status and risk of psychotic disorder or psychotic experiences, measured using the quality assessment tool for observational cohort and cross-sectional studies^1^ (National Institute of Health).

| First author and year of publication | Bolton 2011 | Chakraborty 2011 | Skerrett 2014 | Currier 2015 | Qi 2020 | Post 2021 | Borgogna 2023 | Gevonden 2014  Nemesis I | Gevonden 2014  Nemesis II | Jacob 2021 | Oh 2021 | Oh 2022 | Lu 2023 | Pérez-  Albéniz  2023 |
| --- | --- | --- | --- | --- | --- | --- | --- | --- | --- | --- | --- | --- | --- | --- |
| Clear research question? | yes | yes | no | yes | yes | yes | yes | yes | yes | yes | yes | yes | yes | yes |
| Study population clearly defined? | yes | yes | yes | yes | yes | yes | yes | yes | yes | yes | yes | yes | yes | yes |
| Participation rate > 50%? | yes | yes | n.a. | yes | yes | yes | no | yes | yes | yes | yes | yes | no | yes |
| Groups recruited from same population and uniform eligibility criteria? | yes | yes | yes | yes | yes | yes | yes | yes | yes | yes | yes | no | yes | yes |
| Sample size justification, power description, or variance and effect estimates? | yes | yes | no | no | yes | yes | no | yes | yes | yes | yes | yes | yes | yes |
| Exposure measures reliable and valid? | yes | yes | no | yes | yes | yes | yes | yes | yes | yes | yes | yes | yes | yes |
| Outcome measures reliable and valid? | no | yes | no | yes | yes | yes | no | yes | yes | yes | yes | yes | no | yes |
| Assessors of outcome blind to exposure? | no | no | no | no | no | no | no | no | no | no | no | no | no | no |
| Statistical adjustment for confounders? | yes | yes | no | yes | yes | yes | no | yes | yes | yes | yes | yes | yes | yes |
| Number of positive replies | 7 | 8 | 2 | 5 | 8 | 8 | 4 | 8 | 8 | 8 | 8 | 7 | 6 | 8 |

1. Questions of this tool that concerned aspects of longitudinal course were not used (questions 6, 7, 8, 9, 10, 13).

## Supplementary Table 3. Quality of studies on relationship between LGB-status and risk of psychotic disorder or psychotic experiences, measured using instrument developed by authors.

| First author and year of publication | Bolton  2011 | Chakraborty  2011 | Skerrett  2014 | Currier  2015 | Qi  2020 | Post  2021 | Borgogna  2023 | Gevonden 2014  Nemesis I | Gevonden 2014  Nemesis II | Jacob  2021 | Oh  2021 | Oh  2022 | Lu  2023 | Pérez-Albéniz  2023 |
| --- | --- | --- | --- | --- | --- | --- | --- | --- | --- | --- | --- | --- | --- | --- |
| Validity of context | 2 | 2 | 1 | 1 | 2 | 1 | 2 |  |  |  |  |  |  |  |
| Sample size | 2 | 0 | 0 | 0 | 2 | 2 | 2 |  |  |  |  |  |  |  |
| Assessment sexual orientation | 2 | 2 | 0 | 2 | 0 | 2 | 2 | 2 | 2 | 2 | 2 | 2 | 1 | 2 |
| Assessment psychotic disorder | 0 | 2 | 0 | 0 | 1 | 2 | 1 |  |  |  |  |  |  |  |
| Classification psychotic disorder | 0 | 2 | 0 | 2 | 2 | 2 | 0 |  |  |  |  |  |  |  |
| Interrater reliability psychotic disorder | 0 | 0 | 0 | 0 | 0 | 0 | 0 |  |  |  |  |  |  |  |
| Statistical analysis | 5 | 3 | 2 | 0 | 3 | 3 | 3 | 3 | 3 | 3 | 3 | 3 | 4 | 3 |
| Validity of context (psychotic experiences) |  |  |  |  |  |  |  | 2 | 2 | 2 | 2 | 1 | 1 | 1 |
| Response rate (psychotic experiences) |  |  |  |  |  |  |  | 1 | 1 | 1 | 0 | 0 | 0 | 1 |
| Sample size (psychotic experiences) |  |  |  |  |  |  |  | 1 | 1 | 1 | 1 | 2 | 1 | 0 |
| Quality questionnaire psychotic experiences |  |  |  |  |  |  |  | 2 | 2 | 1 | 2 | 2 | 0 | 1 |
| Total score | 11 | 11 | 3 | 5 | 10 | 12 | 10 | 11 | 11 | 10 | 10 | 10 | 7 | 8 |

## Supplementary Table 4. Statistics from all meta-analyses on the relationship between LGB-status and risk of psychotic disorder or psychotic experiences.

See SupplementaryTable4.xlsx

Table includes pooled ORs, heterogeneity statistics, and publication bias fill-and-trim statistics.

## Supplementary Table 5. Possible mediators of an association between LGB status and psychotic phenomena and the proportions of the effect mediated.

Based on a univariate model (Jacob et al., 2021) or on a multivariate model (Gevonden et al., 2014; Post et al., 2019).

| **First author, year** | **Mediator** | **Proportion of effect mediated (%)** | **B (95% CI)** |
| --- | --- | --- | --- |
| **Psychotic disorder** | | | |
| **Post 2019** | Lifetime discrimination  Childhood trauma  Bullied in adolescence |  | 0.23 (0.07-0.44)  0.12 (0.04-0.25)  0.06 (0.02-0.15) |
| **Psychotic experiences** | | | |
| **Gevonden 2014** | Childhood trauma  Past year discrimination  Childhood bullying  Lifetime cannabis  Lifetime other drugs  Urban living  Living without partner | 8  34  7  3  3  2  11 |  |
| **Jacob 2021** | Marital status  Alcohol dependence  Cannabis use  Loneliness  Social Support  Bullying victimization  Perceived stress  Stressful life events  Common mental disorders  Borderline person. traits  PTSD  Sleep problems | 13.0  6.4  8.4  29.1  10.5  15.9  8.9  25.4  18.1  33.5  8.3  19.1 |  |

# References

Gevonden, M.J., Selten, J.P., Myin-Germeys, I., de Graaf, R., ten Have, M., van Dorsselaer, S., …, Veling, W. (2021). Sexual minority status and psychotic symptoms: findings from the Netherlands Mental Health Survey and Incidence Studies (NEMESIS). *Psychological Medicine, 44*(2):421-33. doi: 10.1017/S0033291713000718.

Jacob, L., Smith, L., McDermott, D., Haro, J.M., Stickley, A., Koyanagi, A. (2021). Relationship between sexual orientation and psychotic experiences in the general population in England. *Psychological Medicine, 51*(1):138-146. doi: 10.1017/S003329171900309X.

Post, D., Veling, W., GROUP investigators (2021). Sexual minority status, social adversity and risk for psychotic disorders-results from the GROUP study. *Psychological Medicine, 51*(5):770-776. doi: 10.1017/S0033291719003726.
